# Enhancing Sleep Quality: The Impact of the “Repose Tao” Pillow with Taopatch^®^ Nanotechnology—A Pilot Study

**DOI:** 10.3390/clockssleep7030032

**Published:** 2025-06-24

**Authors:** Francesca Campoli, Francesca Orofino, Giuseppe Messina, Donatella Di Corrado, Vincenzo Cristian Francavilla

**Affiliations:** 1 Department of Human Sciences and Promotion of the Quality of Life, San Raffaele University, 00166 Rome, Italy; francesca.campoli@uniroma5.it (F.C.); giuseppe.messina@uniroma5.it (G.M.); 2 Department of Sport Sciences, Kore University, Cittadella Universitaria, 94100 Enna, Italy; francesca.orofino@unikore.it; 3 Department of Medicine and Surgery, Kore University, 94100 Enna, Italy; vincenzo.francavilla@unikore.it

**Keywords:** sleep, technological devices, Taopatch, pillow

## Abstract

Background. Sleep disorders are a group of conditions that disrupt normal sleep patterns and are among the most common clinical challenges faced today. An innovative device that employs nanotechnology to deliver beneficial effects on the human body is the Taopatch^®^ (Tao Technologies, Vedelago, Italy). This study aims to assess the effectiveness of such nanotechnology-based devices in improving sleep quality. Methods. This study included only female participants, as a review of the literature indicated that sleep disorders are more prevalent in women than in men. A total of 30 subjects (with a mean age of 44.8 ± 3.44 years) were randomly divided into two groups: an experimental group and a control group. Sleep quality was evaluated three times throughout the study for each participant using the Pittsburgh Sleep Quality Index (PSQI). The Taopatch^®^ devices were applied using a specialized pillow. Results. The experimental group showed significantly better sleep quality (*p* < 0.001) compared to the control group. Conclusions. Our findings suggest that the application of the Taopatch^®^ has a positive impact on sleep quality by optimizing posture, aligning the cervical spine, and promoting muscle relaxation. This device uses advanced nanotechnology to enhance various physiological functions, contributing to better overall well-being.

## 1. Introduction

Sleep quality is influenced by multiple physiological and environmental factors, and physical support of the head and neck during rest is increasingly recognized as a contributor to restorative sleep [1,2].

Disruptions to these rhythms—due to factors like artificial light exposure, irregular sleep schedules, or hormonal fluctuations—can lead to sleep disorders and have broader implications for physical and mental health [3].

Sleep disorders are common and can have significant consequences on patients’ health and overall quality of life. While certain sleep disorders are more difficult to treat, many can be effectively managed with appropriate interventions [4].

The early identification and treatment of these disorders can help recover sleep quality, reduce associated health risks, and enhance daily functioning, contributing to better physical and mental well-being [5].

Sleep disorders refer to a range of conditions that disrupt normal sleep patterns, making them one of the most prevalent clinical issues encountered. Insufficient or poor-quality sleep can negatively impact physical, mental, social, and emotional well-being. These disorders can diminish overall health, safety, and quality of life [6].

The biological system in both animals and humans operates on a cycle that is close to, but not exactly, 24 h. To stay in sync with external environmental time, this system must be reset daily. In most mammals, this process of entrainment is accomplished through regular exposure to light and darkness [7].

Lanzafame proposed that applying low-energy physical forces in specific repetitive frequencies, i.e., physiological rhythms and their potential modulation through external radiant energy, could have profound effects on biological processes [8]. The brain’s molecular clocks are linked to the external world via photoreceptors in the eye, which play a major role in influencing mental and metabolic health [9,10].

New scientific evidence has deepened our understanding of both the crucial role of physiological rhythms and the negative consequences of their disruption [11,12]. These findings have provided insight into how light therapy, also known as photobiomodulation (PBM), has shown promise in enhancing mitochondrial function, reducing oxidative stress, and modulating inflammatory pathways—mechanisms that may help regulate altered physiological rhythms and support the reestablishment of homeostasis in higher biological systems [13,14,15]. Moreover, it has been highlighted how factors such as the timing, intensity, duration, and wavelength of light can impact the human biological clock [16,17,18,19,20].

Light is made up of elementary, massless particles, called photons, which travel through space at the speed of light. These photons interact with the body, making light an essential biological factor for human well-being. The human body’s biological balance is closely linked to light exposure [21,22].

Recently, advances in non-pharmacological sleep interventions have led to increased interest in light-based therapies (near-infrared and ultraviolet light), which are designed to improve the well-being of individuals by enhancing the quality of their sleep [23,24,25].

The Taopatch^®^ (Tao Technologies, Vedelago, Italy) is an innovative nanotechnology device that utilizes light therapy through the use of quantum dots—nanocrystals. These quantum dots convert the energy into near-infrared and ultraviolet light, which can have beneficial effects on the human body. When these quantum dots are excited by body infrared radiation and sunlight, they convert the energy into multiple wavelengths between 200 nm and 600 nm. In humans, this process functions as a form of specific light therapy, providing beneficial and therapeutic effects on the entire body, without releasing any chemical substances [26]. Emerging studies have also explored the interaction between peripheral stimuli—such as light, heat, and touch—and the central nervous system. The vagus nerve, which plays a critical role in parasympathetic regulation and stress modulation, can be influenced by sensory input delivered through the skin [27]. This light is then directed to specific acupuncture points on the body, encouraging it to “remember” how to naturally communicate with itself. This results in numerous health benefits, including improved balance, posture, sleep quality, focus, athletic performance, pain relief, and more [28]. Light therapy helps reduce stress and modulate the endocrine system by regulating hormonal and biological rhythms, such as the body’s sleep–wake cycle [29]. Light also stimulates vitamin D production, which is crucial for bone health, growth, immune defense, and the prevention of chronic, degenerative, and neurological conditions [30].

Currently, the Taopatch is gaining popularity as an innovative device that utilizes nanotechnology to promote beneficial effects on the human body.

While studies have explored the effects of such devices on pain [31], balance and coordination [32], postural control [33], and neurodegenerative diseases [34,35], there are no studies in the scientific literature available regarding the impact of this device on sleep quality, highlighting the need for further investigation in this area.

Based on these arguments, the aim of this study was to evaluate the effectiveness of nanotechnology devices on sleep quality. More specifically, we hypothesized that the integration of Taopatch^®^ nanotechnology into a specialized pillow, called the “Repose Tao Pillow,” would enhance sleep quality by providing optimal support and comfort. Marketed as a tool for improving sleep, we further hypothesized that the experimental group would show superior benefits (e.g., in terms of comfort, relaxation, and overall sleep effectiveness) compared to the control group. For the purposes of this research, the sample was composed exclusively of female participants. This decision was based on a thorough review of the existing literature, which consistently reports a higher incidence of sleep problems and a shorter sleep duration in women compared to men [36,37,38,39].

Another noteworthy finding is the cyclic nature of sleep complaints reported by women [40]. Recent research has uncovered new insights into sleep pathology in women and the crucial role that sex hormones play in regulating sleep and awakenings. Data suggest that during periods of hormonal fluctuations, women are at a higher risk for sleep disorders, including poor sleep quality, sleep deprivation, obstructive sleep apnea, restless legs syndrome, and insomnia [41,42,43].

During childhood and pre-adolescence, there are no significant differences in sleep patterns between boys and girls; however, the main differences begin to emerge with the onset of the first menstrual cycle. With menarche, ovarian function increases, leading to the cyclical production and release of female hormones (estradiol and progesterone) into the bloodstream. These hormones regulate various homeostatic functions, including those related to the cardiovascular, respiratory, and metabolic systems, as well as the sleep–wake cycle [44]. As women approach menopause and during the perimenopausal period, physiological hormonal declines can lead to the onset of sleep disturbances. Normally, sleep regulation is influenced by various physiological processes, including thermoregulation and hormonal rhythms. For instance, the onset of sleep is typically associated with peripheral vasodilation and a corresponding drop in core body temperature, which facilitates rapid sleep initiation. However, in postmenopausal women, this thermoregulatory response may be altered by fluctuations in estrogen and progesterone across different life stages, including the menstrual cycle, pregnancy, and menopause [45].

In summary, the convergence of evidence from chronobiology, hormonal studies, phototherapy, and sensory neuromodulation supports the rationale for exploring the role of nanotechnology-driven light therapy in enhancing sleep quality. However, while the theoretical framework is compelling, empirical data—particularly in the context of sleep—remain limited. Thus, investigating the effects of devices such as the Taopatch^®^ within a study focusing on sleep is both timely and necessary to fill existing gaps in the literature.

## 2. Results

The characteristics of participants are illustrated in Table 1.

Our data reported significantly better sleep quality among participants who used the “Repose Tao” pillow with Taopatch^®^ technology compared to those in the control group. The mean values and standard deviations for each sleep variable across the time points and groups are presented in Table 2.

*Subjective sleep quality*: There was a significant main effect of time on subjective sleep quality (F(_1, 27_) = 21.35, *p* < 0.01, η^2^ = 0.20). Post hoc comparisons revealed a significant reduction in subjective sleep disturbance scores in the experimental group from T_0_ (1.2 ± 0.7) to T_2_ (0.4 ± 0.6; *p* < 0.01). The control group also showed a reduction, though to a lesser extent, from T_0_ (1.4 ± 0.8) to T_2_ (0.8 ± 1.1) (*p* < 0.05).

*Sleep latency*: A significant effect of time was also observed for sleep latency (F(_1, 27_) = 39.06, *p* < 0.01, η^2^ = 0.32). The experimental group demonstrated a reduction from T_0_ (1.1 ± 0.9) to T_2_ (0.5 ± 0.5) (*p* < 0.05). Interestingly, the control group showed an even greater reduction from T_0_ (1.6 ± 0.5) to T_2_ (0.1 ± 0.3) (*p* < 0.01).

*Sleep duration*: A significant change in sleep duration was found across time points (F(_1, 27_) = 15.31, *p* < 0.01, η^2^ = 0.15). The experimental group showed a decrease in reported sleep difficulties from T_0_ (1.3 ± 1.1) to T_2_ (0.9 ± 0.6) (*p* < 0.05), while the control group did not exhibit a significant change.

*Sleep disturbances*: There was a significant time effect for sleep disturbances (F(_1, 27_) = 11.14, *p* < 0.01, η^2^ = 0.12). The experimental group reported lower disturbance scores from T_0_ (1.4 ± 0.5) to T^2^ (0.7 ± 0.5) (*p* < *0.05*), while the control group remained relatively stable.

*Daytime dysfunction*: A strong time effect was found for daytime dysfunction (F(_1, 27_) = 19.11, *p* < 0.01, η^2^ = 0.17). Scores in the experimental group declined from T_0_ (0.7 ± 0.5) to T_2_ (0.4 ± 0.6) (*p* < 0.05), indicating reduced dysfunction, whereas the control group showed an increase from T_0_ (1.4 ± 0.7) to T_2_ (2.0 ± 0) (*p* < 0.05).

*Total PSQI score*: The overall PSQI score showed a highly significant effect of time (F(_1, 27_) = 42.35, *p* < 0.001, η^2^ = 0.39). The experimental group showed a marked reduction in total PSQI scores from T_0_ (6.5 ± 3.9) to T_2_ (3.2 ± 1.9) (*p* < 0.01). The control group also showed a decline in scores from T_0_ (8.7 ± 2.7) to T_2_ (6.0 ± 1.7) (*p* < 0.01), although their scores remained significantly higher than the experimental group at T_2_.

No significant differences were observed between groups in *Habitual Sleep Efficiency* (*p* = 0.82) and *Use of Sleeping Medication* (*p* = 0.10). The Bonferroni post hoc tests confirmed that these changes were statistically significant, especially between T_0_ and T_2_. Finally, the total sleep score was significantly higher in the experimental group, with a very large effect size (*η*^2^ = 0.39).

## 3. Discussion

The purpose of this study was to evaluate the effectiveness of nanotechnology-based devices on sleep quality. Our hypotheses were partially supported by the results, which demonstrated that the “Repose Tao” pillow, incorporating Taopatch^®^ nanotechnology, significantly enhances sleep quality compared to a placebo pillow. The experimental group (real pillow) showed consistent improvements across all sleep dimensions, especially in subjective sleep quality, sleep disturbances, and daytime functioning. The control group showed some improvements, particularly in sleep latency, but in some areas (like daytime dysfunction), their scores were worse. The total sleep score improved much more in the experimental group, with a very large effect size.

These findings suggest that integrating nanotechnology into sleep products—such as the “Repose Tao” pillow—may offer a promising non-pharmacological approach to improving sleep quality. The notable reduction in sleep latency indicates that the “Repose Tao” pillow helps users fall asleep more quickly. This effect is particularly valuable for individuals’ experiencing insomnia or delayed sleep phase syndrome, as reduced sleep latency is closely linked to better overall sleep quality [46,47]. The contoured shape of the “Repose Tao” pillow is specifically designed to support the natural curvature of the cervical spine, thereby reducing neck strain and promoting a more neutral sleeping posture. This ergonomic advantage is especially beneficial for individuals prone to neck stiffness or discomfort—common factors associated with delayed sleep onset. Additionally, poor spinal alignment during sleep has been linked to frequent awakenings caused by discomfort or pressure points [48]. By providing optimal support for the head and neck and ensuring an even weight distribution, the pillow may help reduce the need for frequent repositioning during the night [49].

Beyond its ergonomic design, the inclusion of Taopatch^®^ nanotechnology may also contribute to improved sleep outcomes [50]. The pillow features four strategically positioned Taopatch^®^ strips that interact with key pressure points in the neck and upper head regions. These areas play a crucial role in maintaining spinal alignment and minimizing muscular tension, which may further promote relaxation and support a quicker sleep onset [21,29].

Optimal postural support—such as that provided by the “Repose Tao” pillow—may also help alleviate common sleep disruptors such as tension headaches, neck pain, and micro-awakenings [48]. Sleep efficiency, defined as the ratio of time spent asleep to time spent in bed, is a vital indicator of sleep quality. A higher sleep efficiency is associated with enhanced cognitive performance, mood stability, and overall well-being [51]. Therefore, the combined effect of ergonomic support and nanotechnology in the “Repose Tao” pillow represents a meaningful and innovative intervention for improving both sleep quality and daytime functioning [52].

Moreover, these benefits may be especially relevant for women, who are disproportionately affected by sleep disturbances, including difficulty falling asleep, frequent awakenings, non-restorative sleep, and vivid or disturbing dreams. This heightened vulnerability highlights the need for targeted, non-invasive interventions that address the underlying physiological and neurological mechanisms of disrupted sleep [53,54].

In this context, emerging research suggests that light-based therapies, particularly those delivered through nanotechnology devices, may enhance cellular communication and support systemic regulation in subtle yet meaningful ways [55,56].

### Limitations and Future Research

Despite its promising findings, this study presents several limitations that should be acknowledged. First, the sample was composed exclusively of female participants without diagnosed sleep disorders, which—while this was intentional to reduce variability—limits the generalizability of the results to the broader population, including males, older adults, and individuals with clinically diagnosed sleep disturbances. Second, the sample size was relatively small, which may affect the statistical power and limit the generalizability of the findings. Moreover, sleep was assessed using only subjective self-report measures, which, although validated, may be influenced by individual perception and reporting bias. Future studies should aim to include larger and more diverse samples, as well as incorporate objective sleep measures such as actigraphy or polysomnography to provide a more comprehensive evaluation of sleep patterns. Third, although the study employed a double-blind randomized design to reduce bias, the method of participant selection remains a limitation. Participants were not recruited through standard clinical protocols, and no formal screening procedures were conducted. This non-traditional recruitment approach, while practical for this exploratory study, may affect the representativeness of the sample and limit the generalizability of the results.

Finally, the study relied primarily on subjective measures of sleep quality, such as participant-reported comfort and relaxation, which are inherently influenced by individual perception and may introduce reporting bias. The absence of objective sleep data (e.g., actigraphy, polysomnography, or physiological biomarkers) limits the precision of the conclusions regarding sleep architecture and physiological changes. In fact, a key limitation of this study is the lack of an objective measure of whether the participants complied with the instruction to use the pillow every night. Although the participants were advised to use the device consistently throughout the study period, we did not collect data on their actual usage, which may have introduced variability in the results.

While the present study examined the effects of the Taopatch^®^-integrated “Repose Tao” pillow on sleep quality, the underlying physiological mechanisms remain unclear. Future research could investigate how the nanotechnological components interact with biological systems—such as the nervous or endocrine systems—potentially influencing melatonin production or autonomic nervous system balance. Further studies could assess the efficacy of the Repose Tao pillow in populations with diagnosed sleep disorders, such as insomnia, obstructive sleep apnea, or chronic fatigue syndrome. These groups may particularly benefit from non-pharmacological interventions aimed at improving sleep architecture and reducing pain or discomfort. Long-term investigations are also necessary to determine whether the benefits of the pillow are sustained over time. Furthermore, comparative studies between the “Repose Tao” pillow and other sleep aids—such as ergonomic pillows without nanotechnology—would help clarify its relative effectiveness and further validate its role as a non-pharmacological intervention for improving sleep quality. Finally, sleep quality is influenced by environmental factors such as light exposure, climate, and cultural habits. Multi-site studies conducted across different geographic regions and climates could examine the pillow’s effectiveness under diverse living conditions and social norms.

## 4. Materials and Methods

### 4.1. Participants

The study involved 30 female participants, who were selected from the customers of Manufacture Falomo’s Partners in Italy (https://www.manifatturafalomo.com/pillows/, accessed on 1 January 2020). Each partner was asked to choose two customers to test the “Repose Tao” pillow, following the guidelines provided by Tao Technologies. The inclusion criteria were as follows: participants without sleep disorders (e.g., sleep apnea); who did not use sleep medications; who did not do shift work; who were not new parents (to minimize sleep interruptions); and who had moderate levels of caffeine consumption (an excessive intake could affect their sleep). Five participants who were receiving physical therapy were excluded from the study.

Twenty-five healthy participants, aged between 40 and 50 years (M = 44.8, SD = 3.44), were randomly assigned to two groups: the experimental group (n = 15) and the control group (n = 10). All participants were fully informed about the study protocols prior to its commencement and provided written informed consent for all tests. The study adhered to the ethical guidelines outlined in the Ethical Code of the University of Palermo, as well as the Code of Ethics approved by the General Assembly of the Italian Association of Psychology on 27 March 2015. All procedures were conducted in compliance with the Declaration of Helsinki, and ethical approval was granted by the University Enna Kore Internal Review Board for psychological research (UKE-IRBPSY-11.25.07).

### 4.2. Procedures

The study aimed to understand how sleep quality changed over time and whether these changes differed between those using a real (active) pillow and a sham (placebo) pillow. The study utilized a double-blind randomized design, meaning that neither the participants nor the researchers knew who received the real or sham pillow during the study. Participants were asked to report their sleep quality based on their experiences over the past month, following Tao Technologies’ standard guidelines. Sleep quality was assessed three times for each participant: T_0_ (Baseline), before using the pillow; T_1_, after one week of using the pillow; and T_2_, after 1 month of use. Participants were instructed to use the assigned pillow every night for the duration of the study, which lasted one month. This allowed the researchers to observe the short-term (T_0_ → T_1_) and long-term (T_0_ → T_2_) effects of the Repose Tao pillow on sleep quality.

#### Intervention

**Figure 1:**
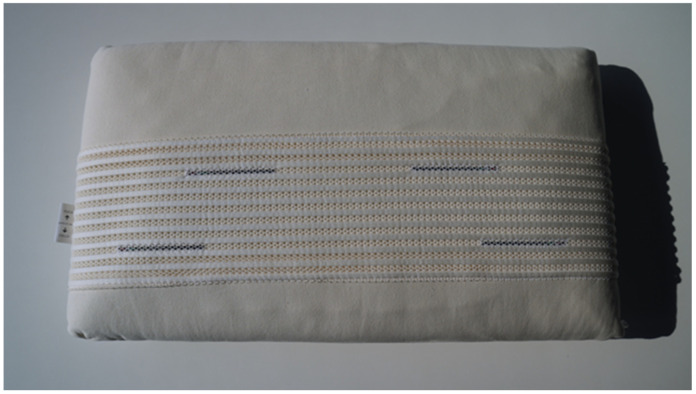
The “Repose Tao” Falomo pillow.

The experimental group (n = 15) used the “Repose Tao” Falomo pillow, which was equipped with active Taopatch^®^ devices. The pillow measures 68 × 40 × 12 cm and weighs 1.8 kg (Figure 1).

**Figure 2:**
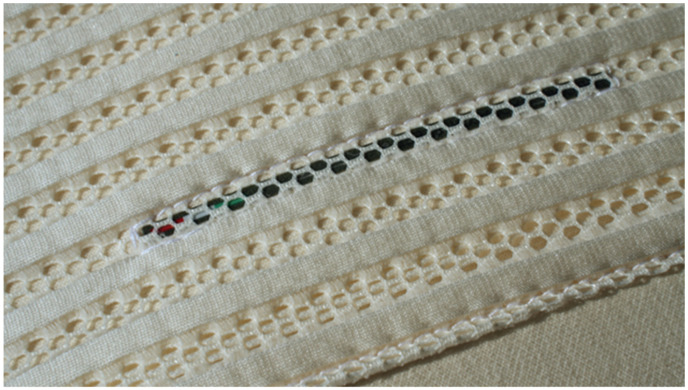
Taopatch^®^ nanotechnology stripes.

Its core is composed of Soia Memory Touch, a soy-based memory foam that provides ergonomic support in various sleeping positions. The upper layer features “Feel” foam, a hydrophilic 3D cotton-based material designed for breathability, elasticity, and comfort, tested in SanoDormire^®^ laboratories. The contoured design is optimized for supine and lateral sleeping, which are among the most common sleep positions. The pillow incorporates four Taopatch^®^ nanotechnology stripes, (10 cm each, <1 mm thick), strategically placed on the upper surface to target key contact areas between the head and pillow, thereby maximizing exposure during sleep (Figure 2).

The arrangement is designed to maximize the interaction time between the integrated nanotechnology and the head during sleep phases. Importantly, the distribution of the nanotechnology stripes is not uniform. To ensure proper usage, a label is placed on one side of the pillow, clearly indicating its correct orientation relative to the user’s body. This labeling ensures the pillow is used correctly and maintains the integrity of the study design. Each Taopatch^®^ device contains nanocrystals that emit low-level light in the infrared and visible spectra, activated by body heat. According to manufacturer specifications, the emitted wavelengths range between 200 nm and 900 nm, with peak intensity in the near-infrared spectrum (~850 nm). The emission is passive, does not require batteries or power sources, and is designed to stimulate local photoreceptive processes. The energy intensity is low, typically in the range of microwatts per square centimeter (μW/cm^2^), ensuring safe, non-invasive interaction over prolonged periods of use.

The control group (n = 10) used a placebo pillow that visually resembled the experimental pillow but was fitted with placebo Taopatch^®^ devices that had no active effects.

Additionally, all participants received training on how to use the pillow correctly to ensure consistency in usage across the study sample.

### 4.3. Measurements

#### Sleep Quality Assessment

Sleep quality was assessed using the Pittsburgh Sleep Quality Index (PSQI) [57], a self-rated questionnaire designed to evaluate sleep quality and disturbances over a one-month period. The PSQI consists of 19 items that generate seven “component” scores: subjective sleep quality, sleep latency, sleep duration, habitual sleep efficiency, sleep disturbances, use of sleeping medication, and daytime dysfunction. Participants are asked to report how frequently they had experienced specific sleep difficulties over the past month and to rate their overall sleep quality. Scores for each item range from 0 to 3, with higher scores indicating more severe sleep disturbances. The PSQI has demonstrated acceptable measures of internal consistency (α = 0.85) and validity. In the present study, the internal consistency was also good (α = 0.83).

### 4.4. Statistical Analysis

The results are expressed as mean ± SD. A repeated measures 3 × 2 ANOVA was conducted to examine the impact of a specialized pillow (Repose Tao pillow) on sleep quality, as measured by the Pittsburgh Sleep Quality Index (PSQI). The factors were time (T_0_ vs. T_1_ vs. T_2_) as within-subjects factor and condition (with the experimental group (EG) using the real pillow and control group (CG) using the sham pillow) as between-subjects factor. Bonferroni correction was applied to control for multiple comparisons. An effect size was used for each analysis with the eta-squared statistic (*η*^2^) to evaluate the practical significance of findings. The ranges for the interpretation of the effect size based on eta-squared indicated a small effect (0.01), moderate effect (0.06), and large effect (0.14) [58]. All statistical analyses were conducted using SPSS version 26 (SPSS Inc., Chicago, IL, USA), with significance set at *p* ≤ 0.05. The results are presented as mean ± SD.

## 5. Conclusions

The present study aimed to evaluate the effectiveness of a nanotechnology-integrated sleep aid—the Repose Tao pillow—in improving sleep quality among healthy female participants. The findings suggest that the use of this specialized pillow may contribute to enhanced sleep experiences, particularly in terms of perceived comfort, relaxation, and overall sleep effectiveness. These results support the potential utility of non-pharmacological, technology-driven interventions in addressing sleep-related concerns, especially in populations that are more vulnerable to sleep disturbances, such as women. The “Repose Tao” pillow significantly improves sleep quality by optimizing posture and supporting proper cervical spine alignment, thereby reducing pressure points that commonly disrupt rest. Its contoured design offers effective support for both supine and lateral sleeping positions, promoting a balanced weight distribution and preventing muscle strain. Additionally, the integration of Taopatch^®^ nanotechnology may enhance muscle relaxation and proprioceptive feedback, contributing to more stable and restorative sleep.

Overall, this study provides a foundation for continued exploration into the application of nanotechnology in sleep health and highlights the importance of innovative, user-friendly solutions for improving sleep quality in targeted populations.

## Figures and Tables

**Table 1:** Participants’ characteristics. Mean (±SD).

	Age (Years)	Height (m)	Weight (kg)
EG	44.3 ± 3.1	1.70 ± 4.85	77.7 ± 7.19
CG	43.4 ± 3.2	1.69 ± 4.36	69.4 ± 6.77

Note: EG is the experimental group and CG is the control group.

**Table 2:** Mean (±SD) for sleep dimensions across session and groups.

Sleep Dimensions		T_0_	T_1_	T_2_	F	*η* ^2^
Subjective Sleep Quality	EG	1.2 ± 0.7	0.8 ± 0.6	0.4 ± 0.6 **	21.35	0.20
	CG	1.4 ± 0.8	0.8 ± 1.1	0.8 ± 1.1 *		
Sleep Latency	EG	1.1 ± 0.9	0.7 ± 0.8	0.5 ± 0.5 *	39.06	0.32
	CG	1.6 ± 0.5	0.7 ± 0.8	0.1 ± 0.3 **		
Sleep Duration	EG	1.3 ± 1.1	0.9 ± 0.7	0.9 ± 0.6 *	15.31	0.15
	CG	2 ± 0.8	1.5 ± 1.1	1.2 ± 0.9		
Sleep Disturbance	EG	1.4 ± 0.5	1 ± 0.3	0.7 ± 0.5 *	11.14	0.12
	CG	1.4 ± 0.7	1 ± 0.8	1.2 ± 0.6		
Daytime Disfunction	EG	0.7 ± 0.5	0.4 ± 0.5	0.4 ± 0.6 *	19.11	0.17
	CG	1.4 ± 0.7	1 ± 0.5	2 ± 0 *		
TOT	EG	6.5 ± 3.9	5.8 ± 2.2	3.2 ± 1.9 **	42.35	0.39
	CG	8.7 ± 2.7	6.2 ± 3	6 ± 1.7 **		

**Notes:** * *p* < 0.05, ** *p* < 0.01. EG = experimental group; CG = control group; T_0_ = baseline, T_1_ = after 1 week; T_2_ = after 1 month.

## Data Availability

The data that support the findings of this study are available from the corresponding author (D.D.C.) upon reasonable request.
